# Quantitative genetic analysis of responses to larval food limitation in a polyphenic butterfly indicates environment- and trait-specific effects

**DOI:** 10.1002/ece3.718

**Published:** 2013-09-02

**Authors:** Marjo Saastamoinen, Jon E Brommer, Paul M Brakefield, Bas J Zwaan

**Affiliations:** 1 Metapopulation Research Group, Department of Biosciences, University of HelsinkiHelsinki, Finland; 2 Department of Biology, University of TurkuTurku, Finland; 3 Section of Evolutionary Biology, Institute of Biology, Leiden UniversityLeiden, The Netherlands; 4 Butterfly Biodiversity Research Group, Department of Zoology, University of CambridgeCambridge, U.K; 5 Laboratory of Genetics, Wageningen UniversityWageningen, The Netherlands

**Keywords:** Environment, heritability, life history, reaction norm, stress

## Abstract

Different components of heritability, including genetic variance (*V*_G_), are influenced by environmental conditions. Here, we assessed phenotypic responses of life-history traits to two different developmental conditions, temperature and food limitation. The former represents an environment that defines seasonal polyphenism in our study organism, the tropical butterfly *Bicyclus anynana*, whereas the latter represents a more unpredictable environment. We quantified heritabilities using restricted maximum likelihood (REML) procedures within an “Information Theoretical” framework in a full-sib design. Whereas development time, pupal mass, and resting metabolic rate showed no genotype-by-environment interaction for genetic variation, for thorax ratio and fat percentage the heritability increased under the cool temperature, dry season environment. Additionally, for fat percentage heritability estimates increased under food limitation. Hence, the traits most intimately related to polyphenism in *B. anynana* show the most environmental-specific heritabilities as well as some indication of cross-environmental genetic correlations. This may reflect a footprint of natural selection and our future research is aimed to uncover the genes and processes involved in this through studying season and condition-dependent gene expression.

## Introduction

Organisms inhabiting heterogeneous and/or seasonal environments often show phenotypic plasticity in which a single genotype yields different phenotypes in response to biotic and/or abiotic aspects of the environment (Pigliucci 2001). The adaptive value of phenotypic plasticity is trait specific; for some traits expressing different phenotypes under heterogeneous environments maximizes fitness, whereas for others maintaining the trait value over a range of conditions (phenotypic canalization) is a more beneficial mechanism (Stearns and Kawecki 1994). In order for phenotypic plasticity to evolve, genetic variation is required in the form of genotype–environment interaction (Via and Lande 1985). Furthermore, understanding the evolutionary potential of organisms requires quantifying the amount of genetic variability expressed for the traits of interest that are under selection. Therefore, to understand the evolution of phenotypic plasticity and to assess the potential for future evolutionary change, it is essential to determine the structure of genetic variation for a suite of traits within and across environments.

Genetic variances depend on allele frequencies and are thus specific to populations and environments (Scheiner 1993; Falconer and Mackay 1996). Heritability of a trait are typically affected by environmental conditions (e.g., Hoffmann and Merilä 1999; Charmantier and Garant 2005; Hallsson and Björklund 2012). This variation in heritability estimates across environmental conditions can be due to changes in the additive genetic variance (*V*_A_) and/or the environmental variance (*V*_E_), and may or may not entail a low genetic correlation between the expressions of the trait across the environments (Hoffmann and Merilä 1999; Charmantier and Garant 2005). A priori predictions about the influence of environmental conditions on the different components of heritability estimates are, however, hampered by discrepancies across studies (e.g., Hoffmann and Parsons 1991; Hoffmann and Merilä 1999; Merilä and Sheldon 1999; Charmantier and Garant 2005). The meta-analyses of studies comparing heritability estimates under favorable versus unfavorable conditions using data from wild populations by Charmantier and Garant (2005) suggested that, in general, estimates of both *V*_A_ and *V*_E_ are decreased under unfavorable conditions. The general trend in experimental research on *Drosophila* and some other insects under laboratory conditions, on the other hand, is that heritability estimates, including *V*_A_, are increased under more stressful conditions (e.g., Hoffmann and Merilä 1999). It has been suggested that some of these differences may be because research under laboratory conditions often uses more extreme and/or more novel environmental stressors (Charmantier and Garant 2005). Importantly, the additive genetic variance has commonly been shown to increase under novel environmental conditions (independent of whether these conditions are favorable or unfavorable), possibly due to expression of genes that have not been under selection in the more common environment (Holloway et al. 1990). Whereas organisms in the wild may typically experience stressful conditions, in the laboratory they do not. Therefore, once adapted to the optimal lab environment, introducing suboptimal conditions may in fact create novel environments. This highlights the importance of choosing a study system that facilitates disentangling these potentially confounding factors. One promising route might be to use a species for which it has been shown that adaptive phenotypic plasticity is an integral part of its natural life history. Using such a study organism, the value and responses of genetic variance estimates of key traits in the predictable environment that drives the phenotypic plasticity and to an unpredictable stressful environment can be determined. Thus, within one biological system, naturally perceived and more unpredictable environments can be contrasted allowing the interpretation of the effects on genetic variation estimates in the light of past and future evolution. Here, we report on this approach using the tropical butterfly *Bicyclus anynana*.

In *B. anynana*, phenotypic plasticity is a crucial component of the life cycle as it lives in highly seasonal environments for rainfall and temperature, and exhibits two very distinct seasonal forms that differ in wing pattern and many other traits (Brakefield et al. 2007). Environmental conditions during development, specifically those related to the thermal environment, are used as a cue for the future environment, and, subsequently, strongly influence hormone dynamics, juvenile growth, and the resulting adult life-history trajectories (e.g., Bauerfeind and Fischer 2005; Saastamoinen et al. 2010; Oostra et al. 2011). Alterations in a suite of life-history and morphological traits in *B. anynana* represent adaptive responses to seasonal differences in reproduction and survival as the wet and dry seasons are associated with favorable and more stressful environmental conditions, respectively (seasonal polyphenism; Brakefield and Larsen 1984; Brakefield et al. 2007, 2009). More specifically, individuals of the wet season form experience warmer ambient temperatures, and as a result have a shorter development time, become smaller as adults, reproduce at faster rate (higher investment to fecundity), and allocate less resources to body maintenance (i.e., fat reserves) resulting in reduced life span compared with the dry season form (e.g., Brakefield and Reitsma 1991; Brakefield and Kesbeke 1997; Pijpe et al. 2006). Dry season forms, on the other hand, tend to experience cooler ambient temperatures in the wild. Ambient temperature during the final larval instar is the main determinant of the two seasonal forms (Oostra et al. 2011). Recently, we have assessed in laboratory experiments how larval resources, which will also vary in nature, influence adult life-history traits in the wet season environment (Saastamoinen et al. 2010). Crucially in the context of our present study, even though developmental nutritional limitation generally reduces body mass and fitness (Bauerfeind and Fischer 2005; Saastamoinen et al. 2010), individuals also changed their body allocation in ways likely to reflect an adaptive response to deteriorating environmental conditions (Van den Heuvel et al. 2013).

Given that we have shown that phenotypic responses in *B. anynana* for a variety of traits work as adaptations allowing individuals to cope more effectively with one or other of the two alternating seasonal environments, responses to food limitation may also be season dependent. For instance, the increased thorax-to-abdomen ratio that occurs in females in response to larval food limitation in the wet season (Saastamoinen et al. 2010) may be less pronounced (or absent) in the dry season, as females in this season already allocate very little to fecundity (i.e., resulting in higher thorax ratio; see Oostra et al. 2011). It is therefore relevant to study plastic responses of the traits, and their interrelationships, under both wet and dry season environments and to determine whether the potential for evolutionary change varies across seasons and environments. In particular the aim of this study was to test the following. First, as genotypes may be more constrained in reaching their potential under harsher environmental conditions (Gebhardt-Henrich and van Noordwijk 1991; Charmantier and Garant 2005), do we observe reduced heritability of performance traits in the laboratory-induced dry season form? Second, as nutritional limitation in the wild is less predictable and hence represents a more novel and less anticipated condition compared with the predictable thermal variation, do we observe increased heritability under conditions of nutritional limitation? Finally, as the levels of additive genetic variances can depend on their relative importance to fitness (Stearns and Kawecki 1994) and the strength or opportunity for selection, are the genetic variance patterns consistent across the phenotypic traits? We implement an information-theoretic approach into a quantitative analysis to arrive at an unbiased estimation of variance components within and across environments that is insensitive to variation in trait means and measurement error. Thus, our study provides comprehensive estimates of genetic variation within and across environments in a species for which adaptive phenotypic plasticity is an integral part of its life history. We will discuss the results in the light of the effects of natural seasonal and unpredictable stressful environments and we will relate our findings to the ecology of *Bicyclus*. We conclude that although significant variation in genetic variance estimates exists between environments and traits, including for the strength of the across-environment genetic correlation, no uniform pattern can be observed. The interpretation of heritability both for this study and in previously published literature will be greatly helped by studying variation in gene expression to allow a direct estimate of the absolute and relative contribution of these genes to the composite estimate that heritability is.

## Materials and Methods

### Study species

*Bicyclus anynana* occurs in tropical and subtropical East Africa and feeds on fallen fruit as an adult. The butterflies used in this experiment originated from the stock population at the Leiden University, which was established in 1988 from over 80 gravid females collected in Malawi. Several hundred butterflies are reared in each generation to sustain high levels of genetic variation (Van't Hof et al. 2005).

To ensure sufficient number of families for the experiment, over 70 mating pairs were established from the stock population. Two- to four-days-old virgin females were randomly mated with 2- to 6-day-old virgin males in an environmental chamber (+27°C, relative humidity 70%, L12:D12). Each female and male was allowed to mate only once. After mating, females were placed individually in a gauze-covered transparent pot with a young maize plant available for oviposition. Females were allowed to lay eggs for 5 days after which the eggs were removed and placed on a petri dish. Twenty-eight families with more than 80 larvae were selected for the present experiment.

### Temperature and diet manipulation during development

**Figure 1:**
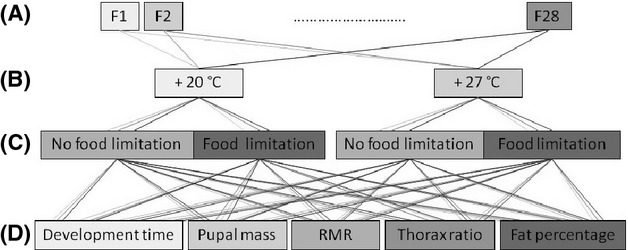
Experimental design: Twenty-eight full-sib families (*A*) were each split and reared under both dry and wet season conditions (*B*; +20 and +27°C, respectively). In the final instar, family groups from both thermal conditions were further split into control and food limitation treatment (*C*). As individuals eclosed six females and six males from each separate family group were assessed for the five life-history traits (*D*).

With the 28 families, we conducted a split brood experiment (Fig. 1) with two thermal environments (seasons), within which we further split each family into two larval food treatments. The first instar larvae in each family were split into two rearing temperatures; +27°C (RH 70%, L12:D12) and +20°C (RH 70%, L12:D12) to mimic the natural environmental conditions during the wet and dry season, respectively (Brakefield 1997). Within each rearing temperature, each family was further divided into two groups of 20 larvae per plant kept inside a sleeve of gauze-like material to ensure nonstressful feeding densities for the family members. Sleeves were checked daily and fresh plants were provided when needed to ensure ad libitum feeding for the larvae.

On the first day of the final, fifth, instar, the larvae within each thermal treatment were further randomly assigned into two larval food treatments: food limitation and control. Equal numbers of individuals within each family were assigned to each treatment. Larvae were transferred individually to a petri dish with either fresh maize leaves (control) or a piece of set agar (1.5 cm^2^ to ensure humidity; food limitation). The larvae were kept in the petri dishes (with or without food) for 2 or 3 days in the warm (wet season) and cooler (dry season) conditions, respectively. One extra day of food limitation was added under the cooler thermal conditions in order to have equal reduction in the body mass due to the food limitation (based on a pilot experiment; M. Saastamoinen, unpubl. data), as larvae, in general, develop more slowly under cooler temperatures. After the 2 or 3 days on a petri dish, individuals were transferred back onto potted maize plants (10 individuals per plant and families separate) with subsequent ad libitum feeding. Larvae from the two different treatment groups and from the different daily cohorts were kept separate to enable development time to be recorded for each individual. Individuals were sexed and weighed 1 day after pupation (Sartorius microbalance GMBH; Sartorius AG, Goettingen, Germany), after which individuals were placed individually in small containers in which the resulting adult butterflies eclosed.

### Life-history traits measured

We assessed development time, pupal mass, resting metabolic rate (RMR), thorax ratio, and fat percentage for three males and three females from each sleeve (i.e., whenever possible six females and six males/family/treatment/season). RMR was measured 1 day after eclosion with a Li-Cor LI-6251 CO_2_ analyzer in a Sable systems respirometer setup with a push-through flow of 100 mL/min. RMR of the individual butterflies was measured in small cylindrical glass containers, which were kept in a temperature-controlled climate chamber (+27°C and +20°C, respectively, for wet and dry season butterflies) for three consecutive measurements of 12 min each. Butterflies were measured in the dark to ensure a resting state of the butterfly. The first CO_2_ reading of each individual was discarded and the second and third readings were averaged before analysis to only include measurements from inactive butterflies. CO_2_ data from the two remaining consecutive measurements were then analyzed using Datacan 5.4 (Sable system, Berlin, Germany). Butterflies were sacrificed after measurement of RMR and the abdomen and thoraces were dried (60°C for 24 h) and weighed (Sartorius microbalance GMBH; Sartorius AG) to assess thorax ratio (thorax dry mass/(thorax dry mass + abdomen dry mass)). For the fat percentage analyses, the dried thoraces and abdomens were submerged in ethyl acetate for 4 days, dried at 60°C for 24 h, and weighed again (Brakefield et al. 2009). The difference between initial dry weight and weight after fat extraction was the absolute fat content. Fat percentage was calculated as the ratio of the absolute fat content to the total dry mass.

### Data analyses

#### Assessment of phenotypic differences in the response to season and food limitation

A linear mixed model approach (SAS version 9.2 for Windows; SAS Institute Inc., Cary, NC) was used to examine the effects of temperature and food treatments on phenotypic variation. In all the analyses, sex was included as a fixed factor, and family and the sleeve nested within a family were included as a random factor. We used backward model selection by starting with a full model for each trait and sequentially eliminating terms with lowest *F*-values until all terms in the model were significant. Development time, fat percentage, and thorax ratio were log transformed so that values were normally distributed. In the analyses of the RMR fat-free dry mass was used as a covariate.

#### Assessment of genotypic differences in the response to season and food limitation

All families were reared under four different environmental conditions mimicking the wet or dry season (temperature), and with or without food limitation. In the analyses, the traits measured were viewed as specific to each of these four environments (character state; Lynch and Walsh 1998). Because we used a full-sib design, we could only estimate genetic (co)variances, which include – apart from additive genetic effects – dominance and maternal effects. Thus, the heritability we estimated must be considered as the upper estimate under the assumptions that dominance and maternal variance components are zero. Family members were reared in different common environments (sleeves), thereby minimizing resemblance across relatives due to rearing effects. Each trait has an environment-specific genetic variance with genetic covariances between the environment-specific trait expressions. Thus, there are in total four genetic variance (*V*_G_) and six genetic covariances (*C*_G_) between all combinations of food limitation (*n* = no stress; *s* = stress) and season (wet and dry defined by thermal conditions). The (co)variances can be summarized as the matrix,


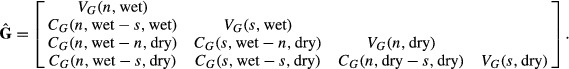
(1)

The genetic covariances can be scaled to a genetic correlation following the standard definition, for example, *r*_G_(*n*,*wet* − *s*, *wet*) = *C*_G_(*n*,*wet* − *s*, *wet*)/√(*V*_G_(*n*,*wet*) × *V*_G_(*s*,*wet*)). Because we estimate compounded genetic (co)variances (see above), this matrix is an approximation of the **G** matrix with additive genetic (co)variances. Whereas the *V*_G_ estimate can be considered as an upper estimate for the additive genetic variance, the *C*_G_ estimate lacks mathematical relationship to the additive genetic covariances because the covariances on the numerous levels (additive genetic, dominance, maternal effect) compounded in *C*_G_ need to align. Hence, our estimates of *C*_G_ and thus of *r*_G_ must be interpret with caution and are not therefore the main focus of this study.

Interactions between genotype and environment (GEI, defined here by the unique combination of season and food limitation) can take two nonmutually exclusive forms (Lynch and Walsh 1998). First, the relative ranking of the breeding values may change between environments. In general, a negative or low genetic covariance between environments indicates that the ranking of genotypes is changed (crossing reaction norms). Second, additive genetic variances may be specific to the environment. However, changes in additive genetic variances across environment are likely to be subjected to scaling, where not only the additive genetic variance changes but also the residual variance (and hence the phenotypic variance). Approaches to study changes in genetic architecture independently from scaling include standardization of variance components with the traits mean (coefficient of variation) or standardization of trait values with their variance (variance standardization; Lynch & Walsh 1998). Here, we follow the latter approach and thereby explore a form of GEI which creates changes in heritability across the four environments. The 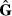 matrix (eq. 1) was estimated by defining the linear mixed model,


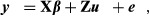
(2)

where ***y*** is a vector of observations on all individuals, ***β*** is a vector of fixed effects, **X** represents a design matrix (of 0s and 1s) relating to the appropriate fixed effects to each individual, ***u*** is a vector of random effects, **Z** is a design matrix relating the appropriate random effects to each individual, and ***e*** is a vector of residual errors. 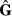 is defined as the matrix for vector **u**, and its elements (the genetic (co)variances) can be estimated by using information on the coefficient of coancestry *Θ*_*ij*_ between individuals *i* and *j*, which is directly obtained from the pedigree. All individuals measured were the descendants of butterflies mated in a full-sib cross. There were 56 base parents with a total of 1206 descendants. The genetic effects in environment *E* (i.e., the combination of food limitation or no food limitation, and dry or wet season conditions experienced) were assumed to be normally distributed with mean of zero (i.e., defined relative to the environment-specific fixed-effect mean) and with an genetic variance of *σ*^*2*^_A,E_. This variance (and the additive genetic covariance between all *E*) was estimated by REML from the variance–covariance matrix of additive genetic effects which is equal to **A***σ*^*2*^, where **A** has elements,


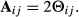
(3)

The fixed-effect structure of equation (2) accounted for variation in age of the butterflies when they entered the final instar and for variation between the sexes. We thus considered all data on both sexes in order to maximize our power to detect changes in genetic variances across environments. By fitting “sex” as a fixed effect, we corrected only for the difference in the mean trait expression between the sexes and thereby assumed that the between-sex genetic correlations for traits did not differ from +1 (no gene-by-sex interaction). In addition, for RMR we included the total fat-free dry weight of the individual as a fixed effect. Residuals were assumed to be heterogeneous (environment specific) and not correlated across environments.

Variances in a linear mixed model are conditional upon the fixed-effect structure. Mixed model phenotypic variance is, in this case, the sum of the REML genetic (including dominance and maternal variances) and residual variances. We incorporated variance scaling by standardizing the raw data prior to analysis to have a REML variance of unity (1) in each environment. This was done by first running a model that only included the fixed effects, where the four residual variances (assumed to be uncorrelated across environments) estimated the environment-specific REML variances. In further analyses, the data were divided by the environment-specific REML standard deviation. By doing so, all trait values become dimensionless (expressed in unit REML phenotypic SD) and the diagonal in equation (1) thereby consisted of the upper estimates of the trait-specific heritabilities.

Given the four environments considered, there are, for each trait, 15 models to consider and, in addition, the null model with residuals only (no heritability). Models were implemented in ASReml (VSN International, Hemel Hempstead, UK), which provides the log likelihood of the mixed model. Because many of the models are not nested, model comparison relied on an information theoretical approach based on the Akaike information criterion (AIC; Akaike 1974; Wagner et al. 1997; Burnham and Anderson 2002). AIC was calculated as −2log(*L*) + 2*K*, where log(*L*) was the model's log likelihood and *K* the number of parameters estimated. All models were ranked in ascending order based on their AIC, where a difference in AIC of more than two compared to the model with the lowest AIC was considered as evidence of deterioration in model fit (Burnham and Anderson 2002). In ASReml, constraining the diagonal of the 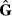 matrix (eq. 1) does not constrain the genetic covariances, which were left unconstrained in all models. We therefore did not consider the covariances or the fixed effects in calculating *K* because these parameters were estimated in all models and hence are factored out when doing model comparisons based on AIC. Thus, we calculated *K* as the number of genetic variances estimates, and *K* ranged from 0 (model with residuals only) to 4 (environmental-specific variances). Akaike weights *w* for model *i* was calculated as *w*_*i*_ = exp(ΔAIC_*i*_)/Σexp(ΔAIC), where ΔAIC_*i*_ is the difference in AIC between model *i* and the top model (i.e., the model with the lowest AIC). Models ranked within two AIC units of the top model were considered as reasonable candidate models (Burnham and Anderson 2002). Because one typically finds some level of support for multiple candidate models, model averaging is advocated to provide more precise estimates (Burnham and Anderson 2002). We model averaged the estimates of the genetic and residual variances across all 15 models, where the model-averaged variance V* was calculated by weighing *V*_*i*_, the variance estimate of model *i*, such that V* = Σ(*V*_i_ × *w*_*i*_) with its model-averaged standard error SE* calculated as 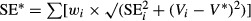 (Burnham and Anderson 2002).

## Results

### Phenotypic variation

**Figure 2:**
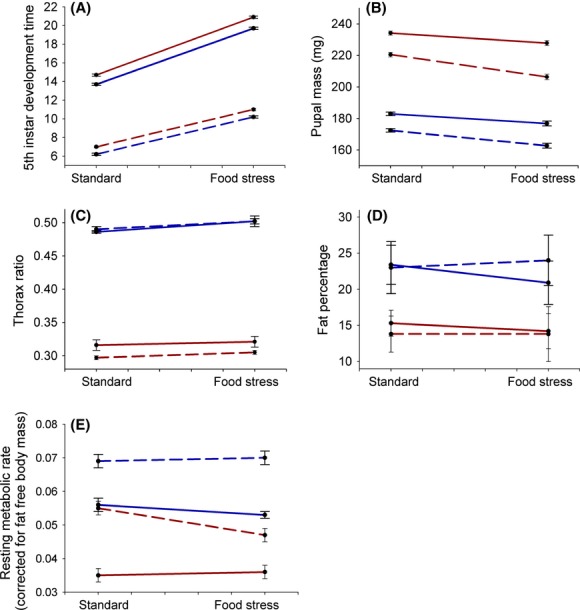
Effects of season (solid and dashed line for dry and wet season, respectively) and larval feeding treatment on development time (*A*), pupal mass (*B*), thorax ratio (*C*), fat percentage (*D*), and resting metabolic rate (RMR; corrected for fat-free dry mass), (*E*) plotted separately for females (red line) and males (blue line). Error bars represent SE of means.

Larvae developed faster and were smaller as pupae under the warmer wet season conditions compared to the cooler dry season conditions, and males developed faster and were smaller as pupae than females (Table 1, Fig. 2A and B). For both of these traits, sex differences were slightly larger in dry season conditions (sex*season interaction; Table 1). Experiencing food limitation during the last instar increased development time and decreased pupal size in both seasons (Table 1, Fig. 2A and B). The effect on development time was larger in the dry season (food limitation*season interaction; Table 1): food-limited larvae took, on average, an extra 4 and 6 days to pupate in wet and dry season conditions, respectively. Importantly, however, the length of the food treatment (i.e., days without food) was also longer in the dry season due to the generally longer development time under these conditions, and hence in both seasons the food-limited individuals needed twice the number of days they had been without food in the experiment to complete development into an adult butterfly (for more details see Material and Methods). The effect of food limitation on pupal mass on the other hand was significantly stronger in the wet season (food limitation*season interaction; Table 1 and Fig. 2B), as the decrease was 4% and 7%, respectively, for dry and wet season.

**Table 1:** Analyses of the effects of season, feeding treatment, and sex on phenotypic traits measured. In all the analyses, family and sleeve nested within family were included as random factors

	df	*F*	*P*
Development time			
Season	1,1084	12642.2	<0.0001
Feeding treatment	1,1084	6440.1	<0.0001
Sex	1,1084	215.2	<0.0001
Season*Sex	1,1084	9.4	0.0022
Season*Feeding treatment	1,1084	124.5	<0.0001
Pupal mass			
Season	1,1079	197.4	<0.0001
Feeding treatment	1,1079	104.0	<0.0001
Sex	1,1079	2795.4	<0.0001
Season*Sex	1,1079	7.6	0.006
Season*Feeding treatment	1,1079	9.1	0.003
Thorax ratio			
Season	1,1090	15.5	<0.0001
Feeding treatment	1,1090	31.5	<0.0001
Sex	1,1090	10361.9	<0.0001
Season*Sex	1,1090	30.8	<0.0001
Fat percentage			
Season	1,1087	1.7	0.187
Feeding treatment	1,1087	13.7	0.0002
Sex	1,1087	1371.8	<0.0001
Season*Sex	1,1087	19.8	<0.0001
Season*Feeding treatment	1,1087	18.8	<0.0001
Resting metabolic rate			
Season	1,1058	301.9	<0.0001
Feeding treatment	1,1058	4.8	0.029
Sex	1,1058	55.9	<0.0001
Fat-free mass	1,1058	139.5	<0.0001

Thorax ratio was higher in males than in females (Table 1, Fig. 2C). The effect of season on thorax ratio was significant (Table 1), however, a significant interaction between sex and season indicated that the relative thorax ratio was only higher in females under dry season conditions (Table 1 and Fig. 2C; see also Oostra et al. 2011). Experiencing stressful food-limited conditions during larval development increased relative allocation to the thorax in both sexes and in both seasons (Table 1, Fig. 2C). The change in the relative allocation to the thorax is caused by a decrease in both abdomen and thorax mass due to food limitation, with a more marked decrease in the former (for females: 10% and 8%; and for males: 11% and 6% decrease, respectively, for abdomen and thorax mass in relation to food limitation).

Males had a higher fat percentage than females (Table 1, Fig. 2D). For males the fat percentage was higher in the wet season compared with the dry season, whereas for females the opposite occurred, and a higher fat percentage was found in this sex under dry season conditions (sex*season interaction; Table 1 and Fig. 2D). Experiencing nutritional limitation decreased fat percentage in general (Table 1). However, there was a significant interaction between the season and effect of stress: food limitation had a negative effect on fat percentage in the dry season, but no effect in the wet season (Table 1 and Fig. 2D).

**Figure 3:**
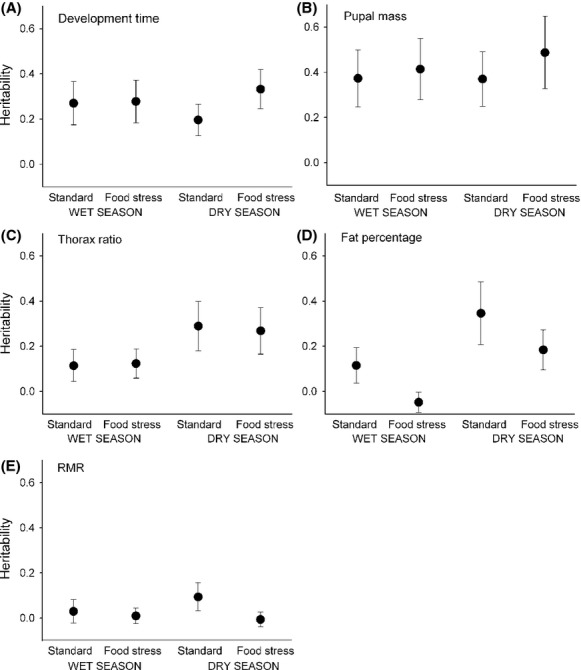
Model-averaged restricted maximum likelihood (REML) estimates of environment-specific upper estimate of heritability with its standard error for development time (*A*), pupal mass (*B*), thorax ratio (*C*), fat percentage (*D*), and resting metabolic rate (RMR, *E*). Four environments are characterized by food stress (standard vs. food limitation) and season (wet season vs. dry season) the larvae experienced during development. Because the data were standardized prior to analysis to have REML variance of unity, the plotted values approximate the environment-specific heritabilities (i.e., *V*_G_ + *V*_R_ ≈ 1 in each environment). Model averaging was performed across all 15 candidate models shown in Table 2. For the traits “RMR” and “fat percentage”, the REML genetic variances were negative in the dry season under food limitation and in the wet season under food limitation, respectively. Further details on the models' fixed effects, residual variances, and estimates are provided in the Supplement.

Heavier individuals had a higher RMR, yet after taking into account the dry body mass, males had a higher RMR than females (Table 1). RMR was additionally higher in individuals reared under wet season conditions (Table 1), but this difference is largely due to the ambient temperatures during the measurements, which were warmer in wet than in the dry season conditions (see Material and Methods). Individuals that had experienced nutritional limitation had a lower RMR than when fed ad libitum throughout their development (Table 1 and Fig. 3E).

### Genetic variation and genotype–environment interactions

The difference in AICs when comparing the model with one constant heritability (model “AAAA”) to the model with residuals only was at least 29 AIC units (Table 2), providing clear evidence that heritable variation was present for all traits in this population. Differences in heritability across the different environments were, however, less clear. Typically, there were several candidate models within two AIC units of the top-ranked model (Table 2). We thus investigated these top models for consistent patterns. For pupal mass and development time, there was no strong statistical evidence of any GEI, as models with equal heritabilities in all environments (model “AAAA”) were within two AIC from the best model (Table 2, Fig. 3).

**Table 2:** Comparison of models allowing for different additive genetic variance structures in the G matrix

Model	log(*L*)	ΔAIC	Weight	Model	log(*L*)	ΔAIC	Weight
Development time				Pupal mass			
**AABA**	−590.00	0.00	0.23	**AAAB**	−546.42	0.00	0.23
**AAAA**	−591.10	0.20	0.18	**AAAA**	−547.49	0.13	0.20
**AAAB**	−590.23	0.47	0.14	**ABAB**	−546.52	0.20	0.19
**ABAB**	−590.40	0.80	0.10	**AABA**	−547.11	1.36	0.06
**ABBA**	−590.51	1.02	0.08	**ABBA**	−547.22	1.60	0.05
**AABC**	−589.66	1.34	0.06	**ABBB**	−547.25	1.66	0.04
**ABCB**	−589.86	1.73	0.04	**ABAC**	−546.29	1.73	0.04
**ABCA**	−589.97	1.94	0.03	**AABB**	−547.32	1.79	0.04
AABB	−591.10	2.20	0.03	**ABBC**	−546.38	1.92	0.03
ABBB	−591.10	2.20	0.03	**AABC**	−546.41	1.97	0.03
ABAA	−591.10	2.20	0.02	ABAA	−547.49	2.13	0.03
ABAC	−590.10	2.21	0.02	ABCB	−546.52	2.20	0.03
ABBC	−590.14	2.30	0.02	ABCA	−547.1	3.34	0.01
ABCD	−589.65	3.31	0.01	ABCC	−547.24	3.62	0.01
ABCC	−591.10	4.20	0.00	ABCD	−546.29	3.72	0.01
res	−626.34	68.76		res	−626.08	155.49	
Thorax ratio				Resting metabolic rate			
**AABB**	−593.90	0.00	0.53	**AABA**	−616.14	0.00	0.31
**AABC**	−593.78	1.76	0.09	**ABAB**	−616.42	0.55	0.18
**ABCC**	−593.89	1.98	0.07	**AAAA**	−617.76	1.23	0.09
AAAA	−595.91	2.03	0.07	**AAAB**	−616.79	1.29	0.08
AABA	−594.99	2.18	0.06	**AABC**	−615.89	1.49	0.07
ABBB	−595.05	2.30	0.05	**ABCB**	−615.93	1.57	0.06
ABAA	−595.36	2.93	0.03	**ABBA**	−617.01	1.73	0.06
ABCB	−594.38	2.96	0.03	ABCA	−616.14	2.00	0.04
ABCD	−593.76	3.71	0.01	ABAC	−616.29	2.30	0.03
AAAB	−595.77	3.74	0.01	AABB	−617.58	2.88	0.02
ABBA	−595.78	3.76	0.01	ABAA	−617.59	2.88	0.02
ABCA	−594.82	3.84	0.01	ABBC	−616.67	3.05	0.01
ABAB	−595.89	3.98	0.01	ABBB	−617.75	3.22	0.01
ABBC	−595.05	4.30	0.01	ABCD	−615.83	3.37	0.01
ABAC	−595.28	4.77	0.00	ABCC	−617.54	4.79	0.00
res	−626.30	61.42		res	−610.60	30.65	
Fat percentage							
**ABCA**	−599.25	0.00	0.51				
**ABCC**	−599.73	0.97	0.19				
**ABCD**	−598.95	1.40	0.13				
AABB	−601.30	2.09	0.06				
ABAA	−601.32	2.15	0.06				
AABC	−600.66	2.82	0.03				
ABAC	−601.21	3.93	0.01				
AABA	−602.29	4.08	0.01				
ABCB	−602.27	6.04	0.00				
ABAB	−604.46	8.41	0.00				
AAAA	−606.18	9.87	0.00				
ABBB	−605.50	10.50	0.00				
ABBA	−605.96	11.42	0.00				
AAAB	−606.13	11.76	0.00				
ABBC	−605.50	12.50	0.00				
res	−625.29	44.75					

Model structure follows the diagonal of equation (1) and letter coding refers to the (dis)similarity of the additive genetic variance for (left to right) no food limitation in the wet season, food limitation in the wet season, no food limitation in the dry season, and food limitation in the dry season. Same letters indicate that the respective additive genetic variances were constrained to be the same. The model with only residuals is indicated as “res”. For each model, the log likelihood (log(*L*)) is presented. All models are ranked in ascending order for their AIC value and, for each model, the difference in AIC value with the model with the lowest AIC value is presented (ΔAIC). All models with an AIC value within 2 units from the model with the lowest AIC value are printed in bold and are all considered as reasonable descriptions of the data.

For thorax ratio, we found good evidence that the dry season increased heritability independently of whether the developing larvae were put under food limitation or not (Table 2, Fig. 3). The covariances between all these environments were strongly positive (Table 3) indicating that ranking of the genotypes was generally maintained. Hence, we find a heritability-increasing genotype – dry season interaction for thorax ratio.

**Table 3:** Model-averaged genetic covariances with their standard error and (in brackets) the scaled covariance (genetic correlation) based on the model-averaged covariances and variances (variances reported in Table S1 and Fig. 3)

	Wet season/No food limitation	Wet season/Food limitation	Dry season/No food limitation
Development time			
Wet season/Food limitation	0.23 ± 0.090 (0.86)		
Dry season/No food limitation	0.13 ± 0.080 (0.56)	0.0042 ± 0.082 (0.018)	
Dry season/Food limitation	0.13 ± 0.090 (0.44)	0.15 ± 0.092 (0.50)	0.10 ± 0.083 (0.50)
Pupal mass			
Wet season/Food limitation	0.35 ± 0.12 (0.88)		
Dry season/No food limitation	0.37 ± 0.12 (0.99)	0.34 ± 0.12 (0.88)	
Dry season/Food limitation	0.39 ± 0.13 (0.91)	0.40 ± 0.14 (0.88)	0.44 ± 0.13 (1.0)
Thorax ratio			
Wet season/Food limitation	0.08 ± 0.06 (0.71)		
Dry season/No food limitation	0.08 ± 0.07 (0.04)	0.15 ± 0.07 (0.77)	
Dry season/Food limitation	0.15 ± 0.07 (0.86)	0.09 ± 0.07 (0.49)	0.26 ± 0.10 (0.92)
RMR			
Wet season/Food limitation	0.02 ± 0.04 (1.1)		
Dry season/No food limitation	0.07 ± 0.05 (1.4)	0.004 ± 0.05 (0.13)	
Dry season/Food limitation	N/A	N/A	N/A
Fat percentage			
Wet season/Food limitation	N/A		
Dry season/No food limitation	0.18 ± 0.08 (0.92)	N/A	
Dry season/Food limitation	0.11 ± 0.07 (0.79)	N/A	0.14 ± 0.08 (0.56)

Covariances and correlations are presented in matrix form between all four environments. Environments are wet season or dry season/food limitation or No food limitation. Note that the variance–covariance matrix was not constrained to be positive definite and some of the scaled covariances therefore fall outside the interval of −1 to 1. For the traits “resting metabolic rate (RMR)” and “fat percentage”, the REML genetic variances was negative in the dry season under food limitation and in the wet season under food limitation, respectively (Fig. 3, Table S1), and hence genetic covariances could thus not be defined (reported here as “N/A”).

The generally low heritability in RMR makes inferences difficult. We find no strong evidence for GEI, as the model with equal heritability in all four environments (model “AAAA”) falls within the top models (Table 2). In addition, the heritability under food limitation in the dry season tended to be negative (Fig. 3, Table S1), indicating difficulties in estimating the environment-specific heritability. The largest heritability is found in the dry season without food limitation (Fig. 3), but the estimate is still very modest (0.094).

The pattern of heritability in fat percentage showed similarities to that of thorax ratio in that there was a clear increase in heritability in the dry season when larvae developed under optimal food conditions (Table 2 and Fig. 3). In addition, there was some evidence of genotype–food limitation interactions for fat percentage in both seasons as many of the top models included this interaction (Table 2, Fig. 3). However, for the wet season this interaction was mainly driven by the estimate of heritability turning negative in the “food limitation/wet” environment (see Table S2 for alternative models where this component is constrained), and this genotype–food limitation interaction was thus not well supported. Fat percentage was the only trait where the genotype–food treatment–season interaction model (model “ABCD” with specific heritability in each environment) was ranked within two AIC from the top model, indicating substantial plasticity in this trait. Although interpretation is hampered by the apparent absence of genetic variance in the wet season under food limitation, the genetic relationships between the environments with non-negative heritability were clearly positive (Table 3), showing that ranking of genotypes for this trait across the environments was generally maintained.

## Discussion

Changes in environmental conditions are known to influence quantitative genetic variation, but there are some discrepancies among studies (e.g., Hoffmann and Merilä 1999; Charmantier and Garant 2005). In this study we specifically aimed to assess phenotypic and genetic (genetic variances and genetic covariances) responses to commonly experienced and predictable environmental fluctuation, and to compare them with responses to a less predictable environmental fluctuation. Thermal conditions during development represented the former conditions as it is an environmental variable that determines the seasonal polyphenism in the study organism *B. anynana* (i.e., wet vs. dry season morph which differs in both wing morphology [data not shown] and life history; e.g., de Jong et al. 2010; Oostra et al. 2011) and hence for which the species has a past history of selection. Food availability during development, on the other hand, was chosen to represent the nonpredictable and hence more novel environmental variable.

### Season-dependent responses to food limitation

As expected, the thermal conditions experienced during larval development produced the two distinct seasonal forms. Hence, individuals under the wet season thermal environment developed faster, were smaller, had a smaller thorax ratio, and lower fat percentage (the last two for females only). The food limitation during development had an equal effect between the two seasons for some traits, whereas for others the responses were season specific. For development time, thorax ratio, and RMR, the responses to food limitation were equal between wet and dry seasonal environments. The significant interaction between season and food limitation for development time is explained by the initial difference of food-limited days (see Material and Methods), as in both cases the development time of the food-limited larvae increased by about twice the number of food-restricted days they experienced as larvae. For thorax ratio, the nonseason-specific response to food limitation is somewhat in contradiction to our recent theoretical model, which showed how an increase in thorax ratio due to poor developmental conditions could be adaptive in the wet season, as only in this season high-quality habitats exist and hence can be located by individuals via dispersal (Van den Heuvel et al. 2013). Thorax ratio can, however, conversely also measure allocation to reproduction, as a low thorax ratio indicates that individuals have allocated more resources into the abdomen (i.e., eggs in females) at the cost of a relatively small thorax. Therefore, the smaller thorax ratios under more optimal conditions may indicate increased investment in reproduction under these conditions, whereas a higher thorax ratio in the dry season may reflect increased allocation to dispersal/food searching capacity. Finally, alteration of metabolism occurs in other organisms (e.g., *Caenorhabditis elegans*; Van Voorhies 2002) in response to rapid environmental change including an absence of food, and presumably decreased RMR under food limitation can enable individuals to cope better under stressful conditions.

The effect of food limitation on pupal mass was larger under the wet season environment. This may indicate that the larvae under dry season conditions are better adapted to compensate for food limitation. This is reasonable as they are more likely to experience such conditions in the wild. Alternatively, nonadaptive explanations may relate to constraints of the thermal environment on physical processes of growth and resources acquisition and allocation (e.g., van der Have and de Jong 1996). The effect of food limitation on fat percentage was similarly season dependent, as food limitation decreased fat percentage only in the dry season. The absolute fat content is higher in dry season butterflies as a result of their larger size, and the relative fat content has been shown to be higher as well over a range of temperatures spanning from the dry to the wet season condition (Oostra et al. 2011; but see our Fig. 2). Fat in the dry season is likely to be used for maintenance and needed for increased stress resistance and increased life span in that season (Pijpe et al. 2007). Our interpretation is that allocation to fat reserves, and hence surviving, under dry season condition is probably maximized, and reduced resources during the larval stage will therefore greatly impact fat content. In contrast, larvae under the wet season conditions are likely to allocate fat to reproduction, and the increased fat allocation under food limitation may be a predictive response to the forthcoming stressful conditions potentially allowing individuals to cope with harsher future conditions (Saastamoinen et al. 2010).

### Changes in heritability across environmental conditions

We used a full-sib design to estimate the upper value of heritability. Our estimate is the upper value because our design cannot partition out genetic dominance variance and maternal effects from the additive genetic variance. We have standardized the variances prior to analyses in order to be sure that changes in the genetic architecture are not merely due to scaling. Variance standardization means that we focus on whether heritability estimates of the traits change across environments. We feel that changes in heritability provide not only an intuitive level to study GEI, but heritabilities also provide the best prediction for the potential of evolutionary change caused by natural selection. The drawback of variance standardization is that an equal heritability across two or more environments does not equate to equal genetic variances, but rather states that differences in variances between environments affect both genetic and environmental variances to the same degree (Houle et al. 2011). In addition, because many non-nested models are compared, we have used model averaging to obtain precise estimates that take into account model uncertainty (Burnham and Anderson 2002).

We find significant heritability for all the life-history traits, although for RMR the estimate was low. In general, the traits we considered appear to show little GEI: only in two of the five traits (thorax ratio and fat percentage) heritabilities changed across thermal environment. For fat percentage there was additionally an indication of GEI across the nutritional environment. The season-specific heritabilities were not in line with our predictions. We predicted, following Charmantier and Garant (2005), reduced heritability in the dry season environment (low ambient temperature), as genotypes may be more constrained under harsher environmental conditions. However, for both thorax ratio and fat percentage, the heritability increased under a dry season environment. Furthermore, we assumed that food limitation would represent an unpredictable environment in our study system, and hence predicted increased heritability under such conditions, especially in the wet season (e.g., Messina and Fry 2003; Dmitriew et al. 2010). Genetic variance did not, in general, react to this change in environmental conditions, with the exception of fat percentage, where – contrary to our prediction – heritability tended to decrease under food-limited conditions.

Apart from causing changes in the additive genetic variance, GEI can also operate by uncoupling the genetic correlations between environments (Falconer and Mackay 1996). Generally, for most of the traits we estimated positive genetic covariances, although our estimates should be interpreted with some caution as our design was not optimal for assessing these (see Material and Methods). Nevertheless, our findings suggest that the main source of GEI in the *B. anynana* system concerns differential expression of genetic variances, rather than low or negative genetic correlations between environments. Hence, it seems likely that the phenotypic values for these traits are governed by the expression and regulation of the same genes and pathways in the different environments (Falconer and Mackay 1996). However, the reported correlations leave room for season-specific variation and this may in part be related to the number of genes contributing to the trait in each season which may explain the variation in heritability between the seasons.

As the levels of genetic variance in a trait are predicted to depend on the relative importance of the trait to fitness (Stearns and Kawecki 1994) and the strength/opportunity for selection, we were interested in whether the heritability patterns were consistent across the phenotypic traits. As stated above, similar season-specific patterns were estimated between thorax ratio and fat percentage. Additionally, for development time and pupal mass, we estimated relatively high heritability, but little variation across environments. Our findings thus underline that generalization of patterns of GEI (Charmantier and Garant 2005) is difficult, especially when dealing with different types of environmental condition (natural vs. experimentally imposed).

### Interpretation of the results in the light of the evolutionary history and ecology of *Bicyclus*

First, we need to address that not finding statistical evidence for an interaction (GEI in our case) may be due to lack of power to detect this interaction. Unfortunately, the power of our quantitative genetic approach is not easily calculated. It should be noted, however, that our design had sufficient power to detect overall significant levels of genetic variances for all traits, including variable levels per trait, from low for RMR to high for pupal mass. This is generally in line with quantitative genetic and life-history theory (Stearns 1992; Roff 1997). The model-averaged estimates of heritability for developmental time and pupal mass in each food/seasonal environment are strikingly close, suggesting that constancy of heritability across the different environments is the most likely cause of not finding GEI in these traits. For RMR, on the other hand, there simply is very little overall genetic variance and thus little “room” for interactions with environment.

Our finding that heritability is higher in the dry season for thorax ratio and fat percentage could potentially be explained by differential selection pressures between the seasons. Reduced larval food sources at the transition between the wet and the dry season and unpredictable resources for the adult in the dry season is part of the environmental variation the species naturally experiences. As thorax ratio and fat percentage are part of the adaptive suit of traits, these traits have been subjected to natural selection. We would therefore expect lower heritability for these traits in a dry season environment. However, we have measured variation in these traits at eclosion, which may not be the time in the life history when these traits contribute the most to fitness, and thus the time when the traits are under the strongest selection. Moreover, even though fat percentage in the dry season is strongly linked with adult starvation resistance in that season, selection on thorax ratio and fat percentage may also operate in the wet season, which is the main reproductive season of the butterfly. As the heritability in a given trait is predicted to depend on its relative contribution to fitness (Stearns and Kawecki 1994) and the strength/opportunity for selection, this would be according to the general predictions. However, as we lack measures of selection operating on life-history traits in the various seasons, in contrast to estimates for wing pattern variation (Brakefield and Frankino 2009), such a scenario remains speculative. Moreover, selection is likely to be complex in the dry season as it must favor an effective and well-timed switch from lipid to glycogen-based energy metabolism (as well as upregulation of the reproductive system at the end of the dry season) and whole-body fat percentage does not include information on these physiological processes. Lastly, the fitness consequences of (slight) deviation from the phenotypes induced by the developmental phenotypic plasticity may be higher in the wet season compared to the dry season for all or some of the traits. Genetic variation may thus be masked and this could involve the activity of molecular chaperones that moderate phenotypic expression of some genetic variants under wet season conditions more than under the dry season conditions (reviewed in Rutherford 2003; Schlichting 2008).

The role of phenotypic plasticity was the main reason for studying genetic variation for life-history traits in this species. It seems evident that in *B. anynana*, the striking plasticity as exhibited in the polyphenism contributes much more to the phenotypic variance across seasons than genetic variation for the traits (see Fig. 2). In a previous study, we have shown that thorax ratio, RMR, and to a lesser extent fat content, but not pupal mass and developmental time, correlate with the hormonal titers that underpin the adaptive phenotypic plasticity (Oostra et al. 2011). It is notable that the latter two traits do not show GEI in this study. This is also true for RMR, but this is likely to be due to the low values of genetic variation for this trait in general. We thus find significant GEI for the traits intimately related to the adaptive phenotypic plasticity, thorax ratio, and fat content. For these traits, within one season the correlation between control and food-limited condition was much higher in the dry season compared to the wet season, which may be related to the lower genetic variance estimates in the latter. More interestingly, cross-environmental correlations between seasons within one food treatment are lower for the control versus the food limitation condition. This may indicate that the processes that contribute to food stress responses are similar across the seasons and in a sense override the processes involved in the polyphenism.

As stated before, the generally positive values of the cross-environmental genetic correlations suggest that expression of similar genes contributes to the genetic variance in both seasons and conditions. This, together with the observed patterns in the cross-environmental correlations, indicates that gene expression studies in the different environments and within families will allow for the identification of the genes and processes that underpin the plasticity and stress responses. This would facilitate the dissection of the composite measure of heritability into its functional components.

## Conclusion

Environmental conditions clearly modulate phenotypic variation of life-history traits in *B. anynana* in addition to genetic variances. Interestingly, the extent of these effects depends on the trait under consideration, as was demonstrated here by very trait-specific patterns in variance components under both seasons and for the two feeding conditions. In three of the five traits examined here, the genetic variances were little affected by changes in season or feeding conditions. For the two traits that show evidence of genotype–environment interactions (thorax ratio and fat percentage), the main pattern is one in which heritability increases under conditions that mark the more adverse (dry) season with generally positive genetic correlations between seasons. This is in contrast to what is expected on the basis of the hypothesis that poor environmental conditions directly constrain the expression of genetic potential and the hypothesis that low cross-environmental genetic correlations underlie changes in additive genetic variance between seasons (Charmantier and Garant 2005). Taken together, this pattern is consistent with low across environmental variation in important traits including developmental time and pupal mass. There is clear seasonal plasticity in traits which may reflect differential allocation of resources (thorax ratio and fat percentage), possibly reflecting season-specific challenges on these allocation decisions and an important role for phenotypic plasticity in setting the trait values. Experimentally induced food limitation had relatively little effect at the genetic level, although some traits showed strong phenotypic responses. The generally high genetic covariances between the seasons and the food limitation manipulations suggest that differences in additive genetic variances are mostly due to differential regulation of the same set of genes; a hypothesis we will be testing this in our future research. Our data and analyses show that studying the environmental dependency of genetic variation and cross-environmental correlations in a species with a legacy of selection for adaptive phenotypic plasticity has clear advantages as it allows the interpretation of the patterns in the light of the species ecology and allows for the formulation of testable hypotheses.

## Supporting Information

**Table S1.** The REML standard deviation (REML SD), coefficients for the fixed effects and model-averaged additive genetic variance (*V*_A_) and residual variances (*V*_R_) for each environment and trait.

**Table S2.** Alternative model ranking when constraining the genetic variance in fat percentage in the wet season under food stress to zero.

## References

[b1] Akaike H (1974). A new look at the statistical model identification. IEEE Trans. Autom. Control.

[b2] Bauerfeind SS, Fischer K (2005). Effects of food stress and density in different life stages on reproduction in a butterfly. Oikos.

[b3] Brakefield PM, Bijlsma R, Loeschke V (1997). Phenotypic plasticity and fluctuating asymmetry as responses to environmental stress in the butterfly *Bicyclus anynana*. Environmental stress, adaptation and evolution.

[b4] Brakefield PM, Frankino WA, Whitman DW, Ananthakrishnan TN (2009). Polyphenisms in Lepidoptera: multidisciplinary approaches to studies of evolution and development. Phenotypic plasticity in insects. Mechanisms and consequences.

[b5] Brakefield PM, Kesbeke F (1997). Genotype–environment interactions for insect growth in constant and fluctuating temperature regimes. Proc. R. Soc. Lond. B.

[b6] Brakefield PM, Larsen TB (1984). The evolutionary significance of dry and wet season forms in some tropical butterflies. Biol. J. Linn. Soc.

[b7] Brakefield PM, Reitsma N (1991). Phenotypic plasticity, seasonal climate and the population biology of *Bicyclus* butterflies (Satyridae) in Malawi. Ecol. Entomol.

[b8] Brakefield PM, Pijpe J, Zwaan BJ (2007). Developmental plasticity and acclimation both contribute to adaptive responses to alternating seasons of plenty and of stress in *Bicyclus* butterflies. J. Biosci.

[b9] Brakefield PM, Beldade P, Zwaan BJ, Behringer RR, Johnson AD, Krumlauf RE (2009). The African butterfly *Bicyclus anynana*: evolutionary genetics and evo-devo. Emerging model organisms: a laboratory manual.

[b10] Burnham KP, Anderson DR (2002). Model selection and multimodel inference: a practical information-theoretic approach.

[b11] Charmantier A, Garant D (2005). Environmental quality and evolutionary potential: lessions from wild populations. Proc. R. Soc. Lond. B.

[b12] Dmitriew C, Blows MW, Rowe L (2010). Ontogenetic change in genetic variance in size depends on growth environment. Am. Nat.

[b13] Falconer DS, Mackay TFC (1996). Quantitative genetics.

[b14] Gebhardt-Henrich SG, van Noordwijk AJ (1991). Nestling growth in the great tit I. Heritability estimates under different environmental conditions. J. Evol. Biol.

[b15] Hallsson LR, Björklund M (2012). Selection in a fluctuating environment leads to decreased genetic variation and facilitates the evolution of phenotypic plasticity. J. Evol. Biol.

[b16] van der Have TM, de Jong G (1996). Adult size in ectotherms: temperature effects on growth and differentiation. J. Theor. Biol.

[b17] Hoffmann AA, Merilä J (1999). Heritabble variation and evolution under favourable and unfavourable conditions. Trends Ecol. Evol.

[b18] Hoffmann AA, Parsons PA (1991). Evolutionary genetics and environmental stress.

[b19] Holloway GJ, Povey SR, Sibly RM (1990). The effect of new environment on adapted genetic architecture. Heredity.

[b20] Houle D, Pélabon C, Wagner GP, Hansen TF (2011). Measurement and meaning in biology. Q. Rev. Biol.

[b21] de Jong MA, Kesbeke FMNH, Brakefield PM, Zwaan BJ (2010). Geographic variation in thermal plasticity of life history and wing pattern in *Bicyclus anynana*. Clim. Res.

[b22] Lynch M, Walsh B (1998). Genetics and analysis of quantitative traits.

[b23] Merilä J, Sheldon BC (1999). Genetic architecture of fitness and nonfitness traits: empirical patterns and development of ideas. Heredity.

[b25] Messina FJ, Fry JD (2003). Environment-dependent reversal of a life history trade-off in the seed beetle *Callosobruchus maculatus*. J. Evol. Biol.

[b26] Oostra V, Invergo MA, de Jong BM, Keskebe F, Wende F, Brakefield PM (2011). Translating environmental gradients into discontinuous reaction norms via hormone signalling in a polyphenic butterfly. Proc. R. Soc. Lond. B.

[b27] Pigliucci M (2001). Phenotypic plasticity: beyond nature and nurture.

[b28] Pijpe J, Fischer K, Brakefield PA, Zwaan BJ (2006). Consequences of artificial selection on pre-adult development for adult lifespan under benign conditions in the butterfly Bicyclus anynana. Mech. Ageing Dev.

[b29] Pijpe J, Brakefield PM, Zwaan BJ (2007). Phenotypic plasticity of starvation resistance in the butterfly *Bicyclus anynana*. Evol. Ecol.

[b30] Roff DA (1997). Evolutionary quantitative genetics.

[b31] Rutherford SL (2003). Between genotype and phenotype: protein chaperones and evolvability. Nat. Rev. Genet.

[b32] Saastamoinen M, Vastenhout D, van der Sterren N, Zwaan BJ, Brakefield PM (2010). Predictive adaptive responses: condition-dependent impact of adult nutrition and flight in the tropical butterfly *Bicyclus anynana*. Am. Nat.

[b33] Scheiner SM (1993). Genetics and evolution of phenotypic plasticity. Annu. Rev. Ecol. Syst.

[b34] Schlichting CD (2008). Hidden reaction norms, cryptic genetic variation, and evolvability. Ann. N. Y. Acad. Sci.

[b35] Stearns SC (1992). The evolution of life histories.

[b36] Stearns SC, Kawecki TJ (1994). Fitness sensitivity and the canalization of fitness components. Evolution.

[b37] Van den Heuvel J, Saastamoinen M, Brakefield PM, Kirkwood TBL, Zwaan BJ, Shanley DP (2013). The predictive adaptive response: modeling the life history evolution of the butterfly, *Bicyclus anynana*, in seasonal environments. Am. Nat.

[b38] Van Voorhies WA (2002). The influence of metabolic rate on longevity in the nematode *Caenorhabditis elegans*. Aging Cell.

[b39] Van't Hof AE, Zwaan BJ, Saccheri IJ, Daly D, Bot ANM, Brakefield PM (2005). Characterization of 28 microsatellite loci for the butterfly *Bicyclus anynana*. Mol. Ecol. Notes.

[b40] Via S, Lande R (1985). Genotype–environment interaction and the evolution of phenotypic plasticity. Evolution.

[b41] Wagner GP, Booth G, Bagheri-Chaichian H (1997). A population genetic theory of canalization. Evolution.

