# Supplementary material for: Quantitative genetic analysis of responses to larval food limitation in a polyphenic butterfly indicates environment- and trait-specific effects
Source: Ecol Evol. 2013 Sep 2;3(10):3576–89. doi: 10.1002/ece3.718 (PMC3797501; doi:10.1002/ece3.718)
Supplement: Supplementary file 1 [file ece30003-3576-SD1.docx]

**Supplementary Table S1.** The REML standard deviation (REML SD), coefficients for the fixed effects and model-averaged additive genetic variance (V_A_) and residual variances (V_R_) for each environment and trait. To correct for scaling effects, the raw data were, prior to analysis, divided by the REML SD such that the REML variance estimated after the fixed effects were accounted for was unity in each combination of season and food stress. Hence, fixed effects and variances reported here are not on the scale of the raw data. Furthermore, variances estimated by the random effects in the model essentially consider the proportion of phenotypic variance due to genetic, dominance ad maternal effects (upper value of heritability *h^2^*) and the proportion of phenotypic variance due to residual (prop res). The values for model-averaged heritability are those plotted in Fig. 3 in the main text.

**————————————————————————————————————————**

Trait/Effect No stress / Wet Stress / Wet No stress / Dry Stress / Dry

**————————————————————————————————————————**

**Pupal mass**

REML SD 0.017123 0.018692 0.014491 0.018561

Intercept 10.1 ± 0.59 11.7 ± 0.63 13.5 ± 0.53 8.58 ± 0.60

Sex (female) 2.81 ± 0.11 2.53 ±0.12 3.58 ±0.11 2.70 ±0.11

Day 5^th^ –0.0031 ±0.033 –0.17 ±0.035 –0.028 ± 0.016 0.029 ± 0.019

V_A_ 0.3726 ± 0.1259 0.4139 ± 0.1358 0.3696 ± 0.1213 0.4870 ± 0.1598

V_R_ 0.653 ± 0.0973 0.620 ± 0.104 0.658 ± 0.0973 0.528 ± 0.112

**Development time**

REML SD 0.937070 1.432131 1.278671 1.371496

Intercept 5.55 ± 0.62 5.84 ± 0.65 9.42 ± 0.57 12.43 ± 0.65

Sex (female) 0.75 ± 0.12 0.57 ±0.12 0.76 ±0.11 0.66 ±0.11

Day 5^th^ 0.055 ±0.034 0.065 ±0.036 0.036 ± 0.018 0.059 ± 0.020

V_A_ 0.270 ± 0.0962 0.278 ± 0.0937 0.196 ± 0.0701 0.332 ± 0.0860

V_R_ 0.747 ± 0.0924 0.7389 ± 0.0957 0.833 ± 0.0955 0.649 ± 0.0967

**Thorax Ratio**

REML SD 0.0390896 0.0354829 0.0208447 0.0290688

Intercept 10.3 ± 0.59 11.7 ± 0.63 22.0 ± 0.55 18.1 ± 0.63

Sex (female) –5.1 ± 0.12 –5.5 ±0.13 –8.2 ±0.11 –6.1 ±0.11

Day 5^th^ 0.12 ±0.033 0.12 ±0.036 0.042 ± 0.017 –0.027 ± 0.020

V_A_ 0.114 ± 0.0710 0.123 ± 0.0642 0.289 ± 0.109 0.268 ± 0.103

V_R_ 0.901 ± 0.0928 0.887 ± 0.0969 0.704 ± 0.0950 0.740 ± 0.0977

**Fat Percentage**

REML SD 0.0376032 0.0614654 0.0288652 0.0321248

Intercept 6.3 ± 0.60 2.6 ± 0.58 8.8 ± 0.57 8.4 ± 0.65

Sex (female) –2.4 ± 0.12 –1.7 ±0.13 –2.8 ±0.11 –2.0 ±0.12

Day 5^th^ –0.010 ±0.033 0.074 ±0.032 –0.022 ± 0.017 –0.062 ± 0.020

V_A_ 0.115 ± 0.0791 –0.0480 ± 0.0462 0.346 ± 0.139 0.184 ± 0.0876

V_R_ 0.897 ± 0.0969 1.048 ± 0.0.102 0.643 ± 0.104 0.824 ± 0.0999

**Resting Metabolic Rate**

REML SD 0.020761 0.0072925 0.018265 0.0061303

Intercept 1.3 ± 0.71 4.8 ± 0.75 3.7 ± 0.74 2.9 ± 0.69

Sex (female) –0.76 ± 0.37 –0.89 ±0.32 –2.75 ±0.48 –1.2 ±0.38

Day 5^th^ –0.014 ±0.035 –0.16 ±0.037 –0.032 ± 0.018 0.033 ± 0.019

Fat Free DW 0.12 ± 0.027 0.12 ± 0.026 0.33 ± 0.038 0.26 ± 0.032

V_A_ 0.0296 ± 0.0523 0.0103 ± 0.0347 0.0944 ± 0.0617 –0.0058 ± 0.0331

V_R_ 0.973 ± 0.0953 0.993 ± 0.0954 0.903 ± 0.0966 1.005 ± 0.093
