# Supplementary material for: Quantitative genetic analysis of responses to larval food limitation in a polyphenic butterfly indicates environment- and trait-specific effects
Source: Ecol Evol. 2013 Sep 2;3(10):3576–89. doi: 10.1002/ece3.718 (PMC3797501; doi:10.1002/ece3.718)
Supplement: Supplementary file 2 [file ece30003-3576-SD2.docx]

**Supplementary Table S2.** Alternative model ranking when constraining the genetic variance in fat percentage in the wet season under food stress to zero. Coding of the models as in Table 2 of the main text, where the genetic variance considered is the second variance term ‘B’, except when it needed to be constrained to zero, which is indicated with ‘0’. Constraining this variance to zero changes the AIC ranking of the top six models in Table 2, where especially the model ‘AABB’ (unequal genetic variance across seasons; equal between food stress treatments) and ‘AABC’ (unequal genetic variances across seasons; equal between food stress treatments in the wet season) are now ranked high. Thus, the support in Table 2 for differences in genetic variance between food treatments in the wet season is largely due to the negative genetic variance estimated under food stress in this season.

| *Fat percentage* | |  |
| --- | --- | --- |
| **Model**  **A0CA** | **AIC**  **-599.92** | **ΔAIC**  **0.00** |
| **AABB** | **-601.30** | **0.76** |
| **A0CC** | **-600.32** | **0.81** |
| **A0CD** | **-599.60** | **1.37** |
| **AABC** | **-600.66** | **1.48** |
| **A0AA** | **-601.90** | **1.96** |
| AABA | -602.29 | 2.74 |
| A0AC | -601.81 | 3.78 |
| ABCB | -602.27 | 4.71 |
| ABAB | -604.46 | 7.08 |
| AAAA | -606.19 | 8.54 |
| ABBB | -605.50 | 9.17 |
| ABBA | -605.96 | 10.09 |
| AAAB | -606.13 | 10.42 |
| ABBC | -605.50 | 11.17 |
| res | -625.29 | 44.75 |
